# Secondary respiratory early and late infections in mechanically ventilated patients with COVID-19

**DOI:** 10.1186/s12879-022-07743-2

**Published:** 2022-09-29

**Authors:** María Elena Ceballos, Carolina Nuñez, Javier Uribe, María Magdalena Vera, Ricardo Castro, Patricia García, Gabriel Arriata, Vicente Gándara, Camila Vargas, Angélica Dominguez, Inés Cerón, Pablo Born, Eduardo Espíndola

**Affiliations:** 1 Infectious Disease Department, Faculty of Medicine, Pontificia Universidad Católica de Chile, Diagonal Paraguay 362, 6th Floor, Santiago, Chile; 2 Internal Medicine Department, Faculty of Medicine, Pontificia Universidad Católica de Chile, Santiago, Chile; 3 Intensive Medicine Department, Faculty of Medicine, Pontificia Universidad Católica de Chile, Santiago, Chile; 4 Clinical Laboratory Department, Faculty of Medicine, Pontificia Universidad Católica de Chile, Santiago, Chile; 5 School of Medicine, Faculty of Medicine, Pontificia Universidad Católica de Chile, Santiago, Chile; 6 Department of Public Health, Faculty of Medicine, Pontificia Universidad Católica de Chile, Santiago, Chile

**Keywords:** Coinfections, Secondary infections, COVID-19, SARS-CoV-2, ICU, Mechanical ventilation

## Abstract

**Background:**

Patients with COVID-19 receiving mechanical ventilation may become aggravated with a secondary respiratory infection. The aim of this study was to describe secondary respiratory infections, their predictive factors, and outcomes in patients with COVID-19 requiring mechanical ventilation.

**Methods:**

A cohort study was carried out in a single tertiary hospital in Santiago, Chile, from 1st June to 31st July 2020. All patients with COVID-19 admitted to the intensive care unit that required mechanical ventilation were included.

**Results:**

A total of 175 patients were enrolled, of which 71 (40.6%) developed at least one secondary respiratory infection during follow-up. Early and late secondary infections were diagnosed in 1.7% and 31.4% respectively. Within late secondary infections, 88% were bacterial, 10% were fungal, and 2% were of viral origin. One-third of isolated bacteria were multidrug-resistant. Bivariate analysis showed that the history of corticosteroids used before admission and the use of dexamethasone during hospitalization were associated with a higher risk of secondary infections (p = 0.041 and p = 0.019 respectively). Multivariate analysis showed that for each additional day of mechanical ventilation, the risk of secondary infection increases 1.1 times (_ad_OR = 1.07; 95% CI 1.02–1.13, p = 0.008)

**Conclusions:**

Patients with COVID-19 admitted to the intensive care unit and requiring mechanical ventilation had a high rate of secondary infections during their hospital stay. The number of days on MV was a risk factor for acquiring secondary respiratory infections.

**Supplementary Information:**

The online version contains supplementary material available at 10.1186/s12879-022-07743-2.

## Introduction

The pandemic, caused by the Severe Acute Respiratory Syndrome Coronavirus 2 (SARS-CoV-2), has reached every country in the world, affecting more than five hundred million people so far [1]. The main clinical manifestation of Coronavirus Disease 2019 (COVID-19) in unvaccinated individuals is an acute upper respiratory infection, which can progress to pneumonia. It has been estimated that between 14 and 20% of pneumonia cases are severe enough to require hospital admission, and 5% of them will require invasive mechanical ventilation (MV) [2, 3].

In other respiratory viral infections, such as influenza, the role of secondary infections and their impact on increasing severity and mortality are well defined, [4]. Nevertheless, this has not yet been confirmed for COVID-19. Available studies have reported different prevalence and prognosis of secondary infections in affected patients [5–12]. In fact, between 7 and 94% of patients admitted with COVID-19 have been reported to develop, at least, one secondary respiratory infection, either on admission or during their hospital stay [5–8]. Of them, approximately 3.5% were considered early secondary infections [13]. The most common responsible pathogens identified were *Staphylococcus aureus*, *Haemophilus influenzae*, *Pseudomonas aeruginosa,* and *Klebsiella pneumoniae*. They were attributed to causing between 7 and 91% of secondary infections [5, 9, 10]. Viral agents, such as *respiratory syncytial virus* (RSV) and influenza, have been reported as causative agents in between 3 and 31% of cases [9, 10, 14]. Among fungal agents, secondary infection due to *Aspergillus sp,* has also gained an important role as co-pathogen; in fact, a new entity called COVID-19 associated pulmonary aspergillosis (CAPA) has been recognized [15].

However, to date, little is known about the characteristics and risk factors of patients with COVID-19 that may contribute to the development of secondary respiratory infections. In the same line, it is not clear what impact a secondary infection could have on major outcomes of these patients. In this study, we aimed to describe the risk factors, the most commonly isolated causative agents for developing secondary respiratory infections, and the clinically relevant outcomes in mechanically ventilated patients with COVID-19.

## Methods

This was a prospective cohort study of all mechanically ventilated patients with severe respiratory failure due to COVID-19, hospitalized from June 1st to July 31st, 2020. Our institution is a tertiary academic hospital (Hospital Clínico UC, Red de Salud UC-CHRISTUS) that has 306 beds and 32 ICU beds. Our ICU increased to 90 beds during the COVID-19 crisis and intensivists, as well as ICU-trained nurses, were deployed to these expanded units to ensure a similar standard of care. Each ICU patient was supervised by, at least, one intensivist, and standardized protocols of MV, sedation, and infection management were established.

Our inclusion criteria were patients over 18 years old requiring invasive MV support for COVID-19-related respiratory failure for at least 24 h. Patients could come from the emergency unit, general ward, intermediate care unit, or were transferred from another hospital.

COVID-19 diagnosis was made by SARS-CoV-2 polymerase chain reaction (PCR) technique obtained from nasopharyngeal samples.

Operational definitions:

- Secondary infection: a secondary respiratory infection from any microorganisms diagnosed concomitant to or after an acute SARS-CoV-2 infection, regardless of whether the patient was on MV or not.
- Early secondary infection: a secondary infection diagnosed during the first days of hospital admission. The 5-day cutoff was determined following previous reports [6, 8].
- Late secondary infection: a secondary infection diagnosed after 5 days from hospital admission. The first positive microbiological test was considered for the determination of early or late secondary infection. In this regard, patients transferred from external centers were excluded from this early/late secondary infection analysis.
- A diagnosis of bacterial secondary respiratory infection (tracheobronchitis or pneumonia) was considered when a patient had clinical deterioration such as purulent secretions, hypoxemia, fever or hemodynamic compromise, and new or progressive chest radiographic infiltrates, both of which are associated with suggestive laboratory and microbiology findings. Only the first positive culture or sample of each patient was considered, and further microbiological study was performed according to hospital availability (see Additional file 1 for details). Specimens were obtained when clinically indicated by attending physicians and processed according to standard microbiological recommendations. No microbiological routine surveillance was established, and bacterial colonization was not included in this analysis. For patients on MV, microbiological testing was performed from an endotracheal aspirate (EA) or bronchoalveolar lavage (BAL) through fiberoptic bronchoscopy; cutoffs of > 10^6^ CFU/mL and > 10^4^ CFU/mL were considered for culture positivity, respectively. In patients weaned from MV, the microbiological study was performed from sputum samples. All positive Filmarray® molecular findings were microbiologically confirmed by cultures.
- Antibiotic resistance of bacteria producing secondary infections was examined by the agar dilution method. Extended spectrum β-lactamases (ESBL) in *Enterobacterales* were detected by CLSI recommended test [16] and carbapenemases produced by Gram negative bacilli were determined through CarbaNP test and immunochromatographic NG-Test Carba 5 (NG Biotech, Guipry, France). Multidrug resistant bacteria (MDR) was determined according to Magiorakos criteria [17].
- Bacteriemia secondary to pulmonary infection was determined by the presence of at least one positive blood culture with the same bacteria identified in the airway 48 h before or after the date of the secondary respiratory infection.
- Multiple secondary infections were determined by the isolation of two or more microorganisms in a significant count in respiratory samples from the same patient during his/her ICU stay.
- ICU-hospital acquired respiratory infection was defined as tracheobronchitis or pneumonia that was not incubating at the time of hospital admission and that presents clinically ≥ 48 h after hospital admission in a non-mechanically ventilated patient.
- Ventilator-associated respiratory infection was defined as tracheobronchitis or pneumonia that developed ≥ 48 h after endotracheal intubation. The patient should be on MV or have been extubated in < 48 h [18]. Because of the difficulty in interpreting new or progressive chest radiographic infiltrates in patients with severe COVID-19 pneumonia, we did not make a distinction between tracheobronchitis and pneumonia.
- Possible, probable, and proven COVID-19 associated pulmonary aspergillosis (CAPA) were defined following ECMM/ISHAM consensus criteria [15]. Other invasive mold infections were defined according to the EORTC/MSGERC criteria [19].
- Cytomegalovirus (CMV) secondary respiratory infection was considered in a patient with a positive CMV PCR in bronchoalveolar lavage (BAL), associated with a positive quantitative CMV PCR in blood and/or a ganciclovir treatment indication by the attending physician.
- Piperacillin–tazobactam, carbapenems, polymyxin E, ceftazidime/avibactam, vancomycin, daptomycin, and linezolid were considered to be broad-spectrum antibiotics.

Demographic and clinical variables were recorded electronically on a structured and previously codified data form using REDCap© tools. We collected demographic data including age, gender, comorbidities, and pharmacological immunosuppression used prior to admission. Clinical data included dates of symptoms’ onset, positive SARS-CoV-2 PCR, hospital and ICU admission, and MV connection. Use of antibiotics and previous and hospital exposure to corticosteroids were also recorded. For each episode of secondary infection, we also recorded the time between hospital admission and secondary infection, symptoms, diagnosis methods, isolated microorganisms, treatments, and outcome. All patients were followed-up until hospital discharge or death to analyze in-hospital, as well as 28-days mortality.

### Statistical analysis

For variables with non-normal distribution, non-parametric tests were used. Accordingly, descriptive statistics are shown as median [interquartile range 25–75] or percentages (%). Mann–Whitney U, Kruskal Wallis, and chi-square tests were used according to variables’ characteristics and distribution. Logistic regression models were fitted for hospital mortality and secondary infection as dependent variables. Explanatory variables were chosen according to bivariate results and their clinical relevance.

A multivariate logistic regression model was adjusted using secondary infection as the dependent variable. Covariables were sex, age, any comorbidity, history of corticosteroids used before admission, use of angiotensin-converting enzyme (ACE) inhibitors before admission, time from first symptoms to hospital admission (days), transfer from another hospital, APACHE II score at admission, in-hospital use of dexamethasone, in-hospital use of methylprednisolone boluses, in-hospital use of tocilizumab, tracheostomy, ICU and hospital stay (days) and MV days. An adjusted odds ratio (_ad_OR) with a 95% confidence interval (CI 95%) was used as an effect measure.

For multivariate analysis, we included dexamethasone, methylprednisolone boluses, and tracheostomy that were used previous to secondary infection onset, and for patients that didn’t develop a secondary infection, that were used at any time during hospitalization.

The case fatality rate was calculated with the formula: (number of deceased patients with a secondary infection/number of total patients with a secondary infection) * 100 and (number of deceased patients without a secondary infection/number of total patients without a secondary infection) * 100.

Data were analyzed with Stata 16 SE (StataCorp, College Station, TX. USA). A two-tailed p-value of < 0.05 was considered statistically significant.

This study was approved by the Scientific Ethical Committee of Health Sciences of the Pontificia Universidad Católica de Chile, ID 200711002. Given its descriptive nature, the committee waived the need for informed consent.

## Results

During the study period, a total of 175 patients with COVID-19 requiring MV were admitted to the ICU. The baseline demographics of study participants are described in Table 1.

**Table 1:** Baseline characteristics of patients hospitalized in ICU with COVID-19

	All patients	No secondary respiratory infection	With secondary respiratory infection	P value
N (%)	175	104 (69.4)	71 (40.6)	
Age, years	62 [54–70]	62 [53–70]	62 [55–71]	0.897
Male, n (%)	125 (71.4)	77 (73.3)	48 (67.6)	0.355
Body Mass Index, kg/m^2^	29 [27–32]	30 [28–33]	27 [26–31]	**0.008**
Any comorbidity, n (%)	130 (74.3)	72 (69.2)	58 (81.7)	0.064
Comorbidities, n	1 [1, 2]	1 [0–2]	2 [1–3]	0.177
Diabetes, n (%)	58 (33.1)	30 (28.8)	28 (39.4)	0.144
Arterial hypertension, n (%)	84 (48.0)	47 (45.2)	37 (52.1)	0.368
Cardiovascular disease, n (%)	15 (8.6)	7 (6.7)	8 (11.3)	0.292
COPD, n (%)	7 (4.0)	3 (2.9)	4 (5.6)	0.298
Asthma, n (%)	3 (1.7)	2 (1.9)	1 (1.4)	0.641
Smoking, n (%)	8 (4.6)	6 (5.8)	2 (2.8)	0.299
Obesity, n (%)	40 (22.9)	25 (23.8)	15 (21.1)	0.652
Cancer, n (%)	11 (6.3)	5 (4.8)	6 (8.5)	0.330
Chronic kidney disease, n (%)	5 (2.8)	4 (3.9)	1 (1.4)	0.342
Chronic liver disease, n (%)	4 (2.3)	2 (1.9)	2 (2.8)	0.698
Autoinmune disease, n (%)	7 (4.0)	4 (3.8)	3 (4.2)	0.900
HIV, n (%)	3 (1.7)	1 (1.0)	2 (2.8)	0.353
Immunosuppressant drugs (before admission), n (%)	4 (2.3)	1 (1.0)	3 (4.2)	0.156
Corticosteroids (before admission), n (%)	8 (4.6)	2 (1.9)	6 (8.5)	**0.041**
ACE inhibitors (before admission), n (%)	18 (10.3)	10 (9.6)	8 (11.3)	0.724
Lymphocytes at admission (cels × 10^3^/μL)	0.62 [0.44–0.92]	0.61 [0.43–0.93]	0.62 [0.45–0.94]	0.424
Time from first symptoms to hospital admission, days	7 [4–9.5]	7 [4–10]	7 [5–8]	0.729
Transfer from another hospital, n (%)	31 (17.7)	19 (18.1)	12 (16.9)	0.793
APACHE II score on admission	15 [10–22]	15 [9–22]	15 [10–22]	0.818
SOFA score on admission	5 [3–8]	5 [3–8]	5 [3–8]	0.987
PaO_2_/FiO_2_ on hospital admission	110 [78–164]	114 [75–165]	110 [82–158]	0.763

Bold indicates statistically significant p value

Data are shown as median [quantiles]. Cardiovascular disease includes congestive heart failure, coronary heart disease, arrhythmias, cardiac devices and stroke. Obesity was defined as a BMI > 30. Cancer includes solid tumor, metastases, leukemia and lymphoma

COPD chronic obstructive pulmonary disease, *ACE* angiotensin-converting enzyme

**Fig. 1:**
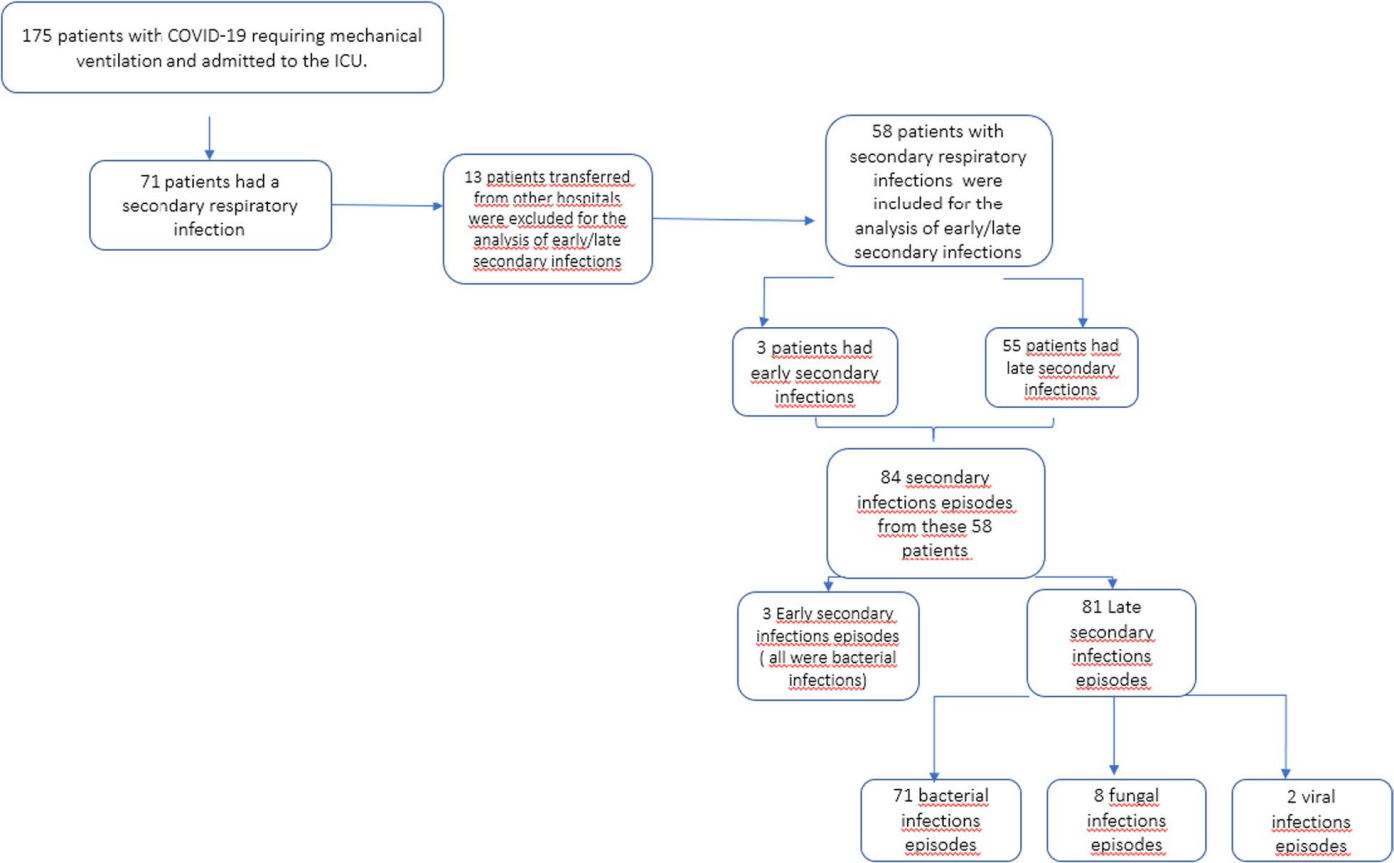
Flow chart

Of the 175 patients, 71 were diagnosed with a secondary respiratory infection (40.6%) either on admission or during their hospital stay. Excluding 13 patients with a secondary infection that were transferred from external centers, we analyzed 58 patients with secondary infections (31.4%). Of them, 3 (5%) patients exhibited early secondary infections and 55 (95%) late secondary infections, representing 1.7% and 31.4% among all patients admitted to ICU, respectively (Fig. 1). Among these 58 patients, there were 84 secondary respiratory infection episodes. Three of them were early and consisted of 3 bacterial infections (one *Staphylococcus aureus* methicillin sensitive, one *Klebsiella pneumoniae,* and one *Pseudomonas aeruginosa*). Late secondary infections consisted in 71 bacterial infections (88%), 8 fungal infections (10%) and 2 (2%) viral infections.

Regarding late secondary bacterial infection episodes, 37 (52.1%) were caused by non-fermentative gram-negative bacilli; 23 (32.4%) by enterobacteriaceae and 11 (15.5%) by gram-positive cocci.

**Fig. 2:**
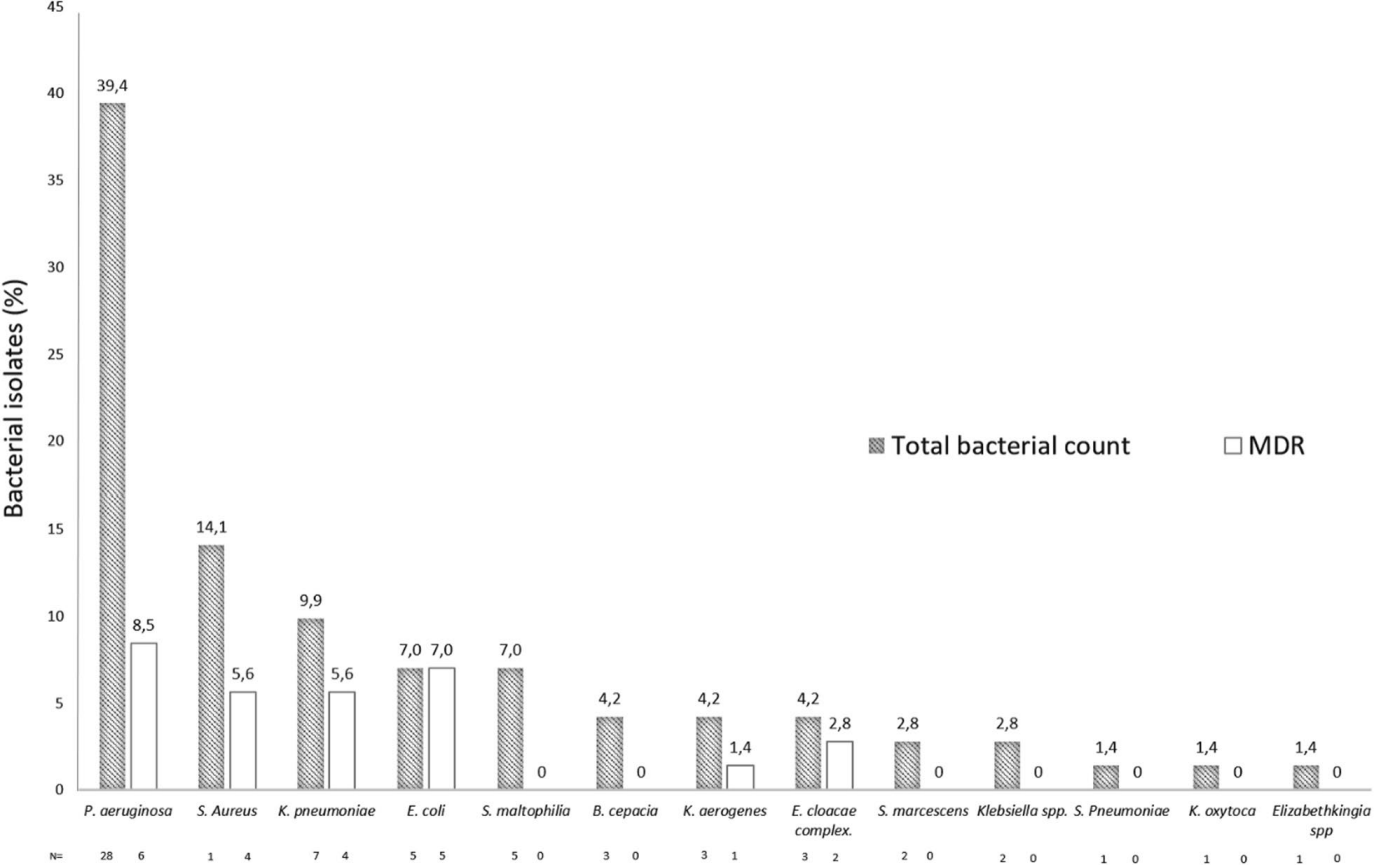
Bacterial isolates in respiratory samples from late bacterial secondary infections. Seventy-one late bacterial secondary infections (excluding patients transferred from another institutions). *MDR* multidrug resistant bacteria; N = number of samples for each bacteria

MDR bacteria were isolated in the 31.0% of late secondary bacterial infections episodes (22/71). The distribution of bacterial species and MDR information among the 71 late secondary bacterial infection episodes are shown in Fig. 2.

Multiple secondary infections were seen in 36 patients (51%). Twenty-four patients (34%) presented 2 secondary infections, 9 (13%) presented 3, and 3 patients presented 4 secondary infections (4%).

Eleven bacterial respiratory infections (15%) were bacteriemic, with a predominance of *Pseudomonas aeruginosa* (55%). Regarding fungal infections, we found one *possible* and 6 *probable* cases of CAPA with isolation of *Aspergillus niger* in one case and *Aspergillus terreus*, *niger*, and *lentulus* in another. We also found one proven case of *Rhizopus* sp. infection. Regarding viruses, there were 2 cases of CMV infections.

Of all patients with secondary infections (71), 60 presented ventilator-associated respiratory infections, and 80% of these ventilator-associated respiratory infections occurred after day 7 of MV. Eleven patients presented ICU-hospital acquired respiratory infections. Among the latter, two were diagnosed before or on the same day of intubation, and 9 were diagnosed 48 h after extubation. Thirty-two patients with a secondary infection required tracheostomy. Of note, 53% of their infections occurred before the intervention and 47% afterwards.

The time between admission and early secondary infections was 2 days (1–4 days) and 24 days (7–80 days) for late secondary infections. Regarding the 3 early secondary infections, two occurred the first 48 h from admission and one occurred on day 4 of admission.

The time from ICU admission to intubation was 4.7 days [IQR 0.1–6.4]. The length of hospital and ICU stay previous to secondary infection development was 19 days [IQR 12–31] and 13 days [IQR 9–20], respectively. The time from secondary infection to death was 17.6 days [IQR 8.8–18.5].

On admission, all patients (with or without secondary infection) were prescribed at least one antibiotic. Within the group of patients with a secondary infection, 65 (91.5%) received antibiotics. Of them, 33 (46.5%) patients received only one antibiotic, 24 (33.8%) received two, and 8 (11%) received 3 to 6 antibiotics. Broad spectrum antibiotics were used in 81.7% of cases. Eight patients received at least one antifungal drug. In our cohort, 15% of all patients (27/175) received at least one dose of tocilizumab or ruxolitinib for the control of SARS-CoV-2-related hyperinflammatory syndrome.

### Comparisons between groups

#### Bivariate analysis

**Fig. 3:**
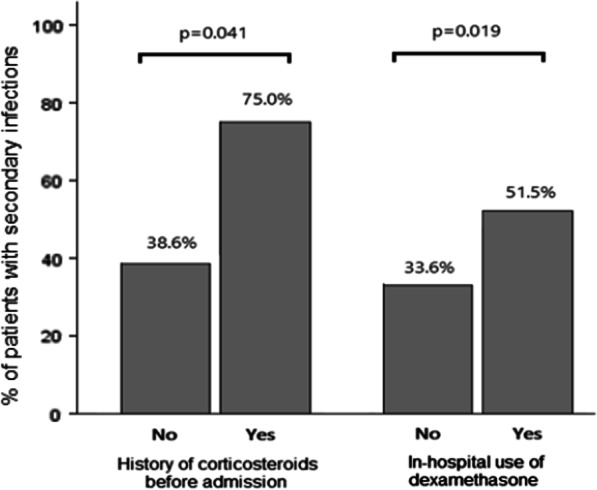
Patients with secondary infection and steroids use

No differences in age or comorbidities were seen between patients with or without a secondary infection, except for body mass index, where a lower BMI was seen in the group of patients with a secondary infection (p = 0.008). Between groups, we did not find differences regarding APACHE or SOFA scores on admission, or with the use of tocilizumab or ruxolitinib. However, the history of corticosteroids used before admission and the use of dexamethasone during hospitalization were associated with a higher risk of secondary infections (p = 0.041 and p = 0.019 respectively). (Tables 1, 2, and Fig. 3).

**Table 2:** Management and outcome of patients hospitalized in ICU with COVID-19

	All patients N = 175	No respiratory secondary infection N = 104	Respiratory secondary infection N = 71	P value
In-hospital use of dexamethasone^a^	68 (38.9)	33 (31.7)	35 (49.3)	**0.019**
In-hospital use of methylprednisolone boluses^a^	72 (42.1)	43 (41.3)	29 (43.3)	0.802
In-hospital use of tocilizumab	22 (12.6)	13 (12.4)	9 (12.7)	0.972
In-hospital use of ruxolitinib	5 (2.9)	3 (2.9)	2 (2.8)	0.979
Awake proning	71 (43.8)	42 (44.2)	29 (43.3)	0.907
Proning in MV	126 (72,8)	72 (70.6)	54 (76.1)	0.426
ECMO	10 (5.7)	4 (3.9)	6 (8.8)	0.183
Tracheostomy^a^	29 (16.7)	13 (12.6)	16 (22.5)	0.085
Renal replacement therapy	24 (14.3)	12 (11.5)	12 (16.9)	0.311
Vasoactive drugs	133 (77.3)	68 (67.3)	65 (91.5)	**< 0.001**
ICU re-admission	5 (2.9)	1 (1)	4 (5.6)	0.068
Length of prone position, days	6 [3–10]	5 [2–9]	6 [3–11]	0.221
Length hospital stay, days	33 [21–52]	30 [20–44]	46 [32–76]	**< 0.0001**
Length ICU stay, days	18 [9–33]	16 [10–23]	26 [15–51]	**< 0.0001**
Length MV, days	14 [9–30]	13 [9–22]	23 [13–48]	**< 0.0001**
Time from admission to death, days	25 [19–36]	21 [16–30]	33 [22–47]	**0.0062**
Death at 28 days	31 (17.7)	20 (19)	11 (15.5)	0.526
Death in ICU	54 (30.9)	30 (28.8)	24 (33.8)	0.486
Case fatality rate	–	30/100	34/100	0.485

Bold indicates statistically significant p value

Data are shown as median [quantiles]or n (%). Methylprednisolone boluses were defined as greater than or equal to 125 mg in 3 occasions

*MV* mechanical ventilation, *ECMO* extracorporeal membrane oxygenation, *ICU* intensive care unit

^a^That were used previous to secondary infection

The occurrence of secondary infections was significantly associated with a higher need for vasoactive drug use, longer ICU and hospital length of stay, and MV requirement than patients with no secondary infection. No significant differences in ICU and 28-day mortality were seen between patients with or without secondary infection.

#### Multivariate analysis

On multivariate analysis, the only variable that was associated with respiratory secondary infections was days in MV (_ad_OR = 1.07; 95% CI 1.02–1.13, p = 0.008) (Additional file 2: Table S2).

## Discussion

In this cohort study, we found that a high rate of patients with SARS-CoV-2 developed a secondary respiratory infection during their ICU stay. The vast majority presented late secondary infections, and a high proportion had more than one secondary infection episode. The use of corticosteroids before admission, as well as in-hospital use of dexamethasone, were associated with a higher risk of secondary infections in the bivariate analysis, but not in the multivariate analysis. The number of days in MV was independently associated with a significantly higher risk of secondary infections.

As shown, more than forty percent of our patients developed at least one secondary respiratory infection during their ICU stay. This is higher than previously reported for COVID-19 [9, 20, 21] but similar to the proportion of secondary infections reported in post influenza ICU patients [22, 23]. The incidence of early vs late secondary infection was similar to other studies, where late secondary infections were predominant [5, 6, 13, 24]. This is to be expected since patients with COVID-19 usually have long hospital stays, making them more susceptible to hospital-acquired infections. Importantly, more than half of our patients with a secondary infection developed multiple secondary infection episodes, probably associated with a longer length of stay. The bacterial resistance prevalence rate is higher than the findings reported by García-Vidal [5]

but lower than the one reported by Li et al. [25], which was over 50%.

The most common bacterial agent in late secondary infections in our cohort was *Pseudomonas aeruginosa*. Earlier findings in patients with COVID-19 describe the same agent as predominant [6, 7]. Nevertheless, other studies show enterobacteriae or *Staphylococcus aureus* as the most common agent in secondary respiratory infection [26, 27]. The latter reflects differences in local microbiology and the importance of knowing this information to make an appropriate first choice of an antimicrobial when indicating an empirical regimen.

We found cases of fungal infections in apparently immunocompetent patients. Nevertheless, other risk factors for secondary fungal infection such as broad-spectrum antibiotic use, corticosteroid use, or prolonged ICU stay may have contributed to these secondary infections [28]. Invasive fungal infections have been reported in ICU patients with COVID-19 [6, 7, 29, 30], similar to what was previously found in influenza [31]. We did not find community-acquired seasonal viruses as secondary infections, even considering that the recruitment of patients was during our winter season. In contrast to preceding years, during the first wave of COVID-19 in our country, classical respiratory viruses were displaced by SARS-CoV-2. The only viral secondary infections we found were due to CMV, which can be explained because of COVID-19 immunosuppression, and the concomitant use of systemic steroids.

In our center, tocilizumab or ruxolitinib were indicated to patients with high suspicion of having a hyperinflammatory syndrome associated with COVID-19. We did not find an association between the use of these drugs and higher secondary infection rates, results that are coherent with other reports [5, 7, 26]. Conversely, patients with chronic use of corticosteroids before admission or patients exposed to dexamethasone in hospital because of COVID-19 developed more secondary infections in bivariate analyses than patients that were not exposed. There are conflicting results on this association; some studies have reported a direct one [5, 26, 32] while others have not [7]. Our study was done before the findings of the RECOVERY trial [33] so we could compare patients with and without steroids. Systemic corticosteroids for the treatment of COVID-19 patients are now broadly used based on studies that showed their efficacy in preventing invasive mechanical ventilation and reducing 28-day mortality for COVID-19 severe cases [33, 34]. However, their impact on immunity, the risk of associated infections, and their ultimate effect on outcomes are still unclear [34].

The fact that both patients with and without secondary infection in our cohort used antibiotics could be explained by the application of local hospital guidelines that initially recommended their prescription for all patients with severe pneumonia. A high frequency of antibiotic exposure previous to a secondary infection development has been previously reported [26]. Considering that a minority of our patients admitted to the ICU presented an early secondary infection, in line with other studies [5, 24, 35, 36], our findings support a more restrictive use of antibiotic therapy on admission and must be limited to suspected or confirmed bacterial infections in these patients.

Despite the high secondary infection rate, ICU mortality in our cohort was similar to other COVID-19 ICU reports [20, 21]. In patients with secondary infections, a higher rate of survival than in our study has been reported, however, these studies included non-ICU patients [5, 7]. We did not find a correlation between secondary infections and mortality. However, we found an association between secondary infections and the use of vasopressors, longer ICU and hospital stay, and longer MV time. The nature of this study does not allow us to establish if these findings are a cause or a consequence of secondary infections. Some studies suggest a higher risk of mortality in patients with a secondary infection [5, 9, 24, 37], but these findings are in contradiction with other publications [6, 11]. More studies are needed to answer this question, which is crucial to determine the real clinical relevance of secondary infections in the COVID-19 pandemic.

Our study has some limitations. First, it was conducted at a single center, thus our microbiological results cannot be generalized to other institutions. Additionally, interpretation of our findings might be limited by the sample size. In particular, the history of corticosteroids used before admission and in-hospital use of dexamethasone could have shown different results in multivariate analysis with a larger sample size. Third, the nature of the study does not allow us to determine causality between secondary infections and worst outcomes such as longer hospital or ICU stay. Despite these limitations, our study has important strengths, such as an extensive microbiological study, antimicrobial resistance, and the comparison with a group of patients with no secondary infection.

## Conclusions

In a cohort of critically ill mechanically ventilated patients with COVID-19, there was a high rate of secondary infections. The number of days on MV was a significant risk factor for acquiring secondary respiratory infections.

## Supplementary Information

**Additional file 1: Table S1.** Microbiological investigation available in our center.

**Additional file 2: Table S2.** Multivariate logistic regression for secondary infection.

## Data Availability

The datasets supporting the conclusions of this article are included within the article and its additional files.

## Abbreviations

MV: Mechanical ventilation
COVID-19: Coronavirus disease 2019
ICU: Intensive care unit
SARS-CoV-2: Severe Acute Respiratory Syndrome Coronavirus 2
VRS: Respiratory syncytial virus
CAPA: COVID-19 associated pulmonary aspergillosis
PCR: Polymerase chain reaction
ESBL: Extended spectrum β-lactamases
CMV: Cytomegalovirus
BAL: Bronchoalveolar lavage
X-ray: Chest radiography
CT: Chest computed tomography
_ad_OR: Adjusted Odds ratio
ACE: Angiotensin-converting enzyme
