# Supplementary material for: Secondary respiratory early and late infections in mechanically ventilated patients with COVID-19
Source: BMC Infect Dis. 2022 Sep 29;22:760. doi: 10.1186/s12879-022-07743-2 (PMC9521562; doi:10.1186/s12879-022-07743-2)
Supplement: Supplementary file 1 — Additional file 1: Table S1. Microbiological investigation available in our center. [file 12879_2022_7743_MOESM1_ESM.docx]

**Table** S**1**

Microbiological investigation available in our center

| **Sample** | **Test** |
| --- | --- |
| Sputum | Gram stain  Baciloscopy  Calcofluor stain  Kinyoun stain  Aerobic and anaerobic bacterial culture  Fungal culture  *Mycobacterium tuberculosis* culture  *Pneumocystis Jirovecii* direct immunofluorescence assay  *Pneumocystis Jirovecii* PCR  FilmArray® Pneumonia Panel  FilmArray® Respiratory Panel  Luminex RPP Respiratory pathogens  Qualitative Cytomegalovirus PCR  Galactomannan |
| Endotracheal aspirate (EA) |  |
| Bronchioalveolar lavage (BAL) |  |
| Nasopharyngeal swabs | Cobas® Influenza A/B  FilmArray®, Respiratory Panel  Luminex RPP Respiratory pathogens |
| Urine | Urine antigen *Legionella pneumophila*  Urine antigen *Streptococcus pneumoniae* |
| Blood | Galactomannan  Quantitative Cytomegalovirus PCR |
