# Supplementary material for: Secondary respiratory early and late infections in mechanically ventilated patients with COVID-19
Source: BMC Infect Dis. 2022 Sep 29;22:760. doi: 10.1186/s12879-022-07743-2 (PMC9521562; doi:10.1186/s12879-022-07743-2)
Supplement: Supplementary file 2 — Additional file 2: Table S2. Multivariate logistic regression for secondary infection. [file 12879_2022_7743_MOESM2_ESM.docx]

| Table S2. Multivariate logistic regression for secondary infection. | | | | |
| --- | --- | --- | --- | --- |
|  | Sig. | Exp(B) | 95%CI | |
|  |  |  | Lower bound | Upper bound |
| Male sex | 0.317 | 1.597 | 0.638 | 3.997 |
| Age (years) | 0.787 | 0.995 | 0.963 | 1.029 |
| Any comorbidity | 0.063 | 3.215 | 0.939 | 11.011 |
| Use of corticosteroids before admission | 0.079 | 5.878 | 0.815 | 42.376 |
| ACE inhibitors before admission | 0.698 | 0.768 | 0.203 | 2.910 |
| Time from first symptoms to hospital admission | 0.671 | 1.017 | 0.941 | 1.100 |
| Transfer from another hospital | 0.449 | 0.602 | 0.162 | 2.238 |
| Apache II score | 0.447 | 0.976 | 0.918 | 1.039 |
| In-hospital use of dexamethasone previous to secondary infection | 0.289 | 1.605 | 0.670 | 3.849 |
| In-hospital use of methylprednisone boluses previous to secondary infection | 0.592 | 0.797 | 0.347 | 1.831 |
| Tracheostomy previous to secondary infection | 0.239 | 0.433 | 0.108 | 1.743 |
| In hospital use of tocilizumab | 0.565 | 0.698 | 0.205 | 2.375 |
| Length of ICU stay (days) | 0.260 | 0.974 | 0.931 | 1.019 |
| Length of hospital stay (days) | 0.179 | 1.019 | 0.992 | 1.047 |
| Days of mechanical ventilation | 0.008 | 1.072 | 1.018 | 1.129 |
| Intercept | 0.031 | 0.068 |  |  |
| Sig.: p value; Exp(B): odds ratio; 95%CI: 95% confidence interval for the odds ratio. | | |  |  |
